# The Impact of Hospital Level of Care on the Management of Acute Cholecystitis: a Population-Based Study

**DOI:** 10.1007/s11605-022-05471-1

**Published:** 2022-10-17

**Authors:** Lisa Lindqvist, Andreas Andersson, Johanna Österberg, Gabriel Sandblom, Oskar Hemmingsson, Pär Nordin, Lars Enochsson

**Affiliations:** 1 Department of Surgical and Perioperative Sciences, Surgery, Umeå University, 901 87 Umea, Sweden; 2 Department of Clinical Sciences, Intervention and Technology (CLINTEC), Karolinska Institute, Stockholm, Sweden; 3 Department of Surgery, Mora Hospital, Mora, Sweden; 4 Department of Clinical Science and Education Södersjukhuset, Karolinska Institute, Stockholm, Sweden; 5 Department of Surgery, Södersjukhuset, Stockholm, Sweden; 6 Department of Surgery, Sunderby Hospital, Lulea, Sweden

**Keywords:** Acute cholecystitis, Hospital level of care, Tertiary referral centers, National registry, Surgery

## Abstract

**Background:**

The organization of healthcare could have an impact on the outcome of patients treated for acute cholecystitis (AC). The aim of this study was to analyze the way in which patients with AC are managed relative to the level of care by the treating hospital.

**Methods:**

Data were collected from the Swedish Register for Gallstone Surgery and ERCP (GallRiks). Cholecystectomies between 2010 and 2019 were included. The inclusion criterion was acute cholecystectomy in patients with AC operated at either tertiary referral centers (TRCs) or regional hospitals.

**Results:**

A total of 24,194 cholecystectomies with AC met the inclusion criterion. The time between admission and acute surgery was significantly elongated at TRCs compared with regional hospitals (2.2 ± 1.7 days vs. 1.6 ± 1.4 days, mean ± SD; *p* < 0.0001). Patients with a history of AC were more frequent at TRC (10.1% vs. 8.9%, *p* < 0.0056) and had a higher adverse event rate compared with those at regional hospitals (OR 1.61; CI 1.40–1.84, *p* < 0.0001).

Surprisingly, an increased number of hospital beds correlated slightly with an increased number of days between admission and surgery (*R*^2^ = 0.132; *p* = 0.0075).

**Conclusion:**

Compared with regional hospitals, patients with AC had to wait longer at TRCs before surgery. A history of AC significantly increased the risk of adverse events. These findings indicate that logistic and organizational aspects of hospital care may affect the management of patients with AC. However, whether these findings can be generalized to healthcare organizations outside Sweden requires further investigation.

## Introduction

Acute cholecystitis (AC) is the most common complication of gallstone disease, affecting 20% of patients admitted for biliary tract disease.^1,2^ Several studies on the optimal time to treat ongoing AC have reported that early laparoscopic cholecystectomy (LC) performed within the first week decreases the overall morbidity and shortens the total length of stay compared with delayed LC.^3,4^ Recent register studies have also indicated that cholecystectomy within 2 days of admission lowers the rate of both intra- and postoperative adverse events and conversion rates.^5,6^

Even though the safest management of gallstone disease, in particular AC, is well known, the treatment and outcome differ depending on where the operation is performed, suggesting that the organization of healthcare is an important factor. A recent study comparing the outcomes of cholecystectomy in different Swedish regions showed large regional differences regarding the treatment of gallstone disease and the postoperative outcome.^7^ These differences did not correlate with population density but rather with differences in surgical management. Furthermore, recent studies indicate that the chance of undergoing a cholecystectomy soon after admission for AC appears to be more dependent on healthcare organization than the individual needs of the patient.^8,9^

We aimed to determine if the level of care of the treating hospital might play a role in how soon after admission patients with ongoing AC undergo cholecystectomies in Sweden.

## Methods

### Study Design

**Fig. 1:**
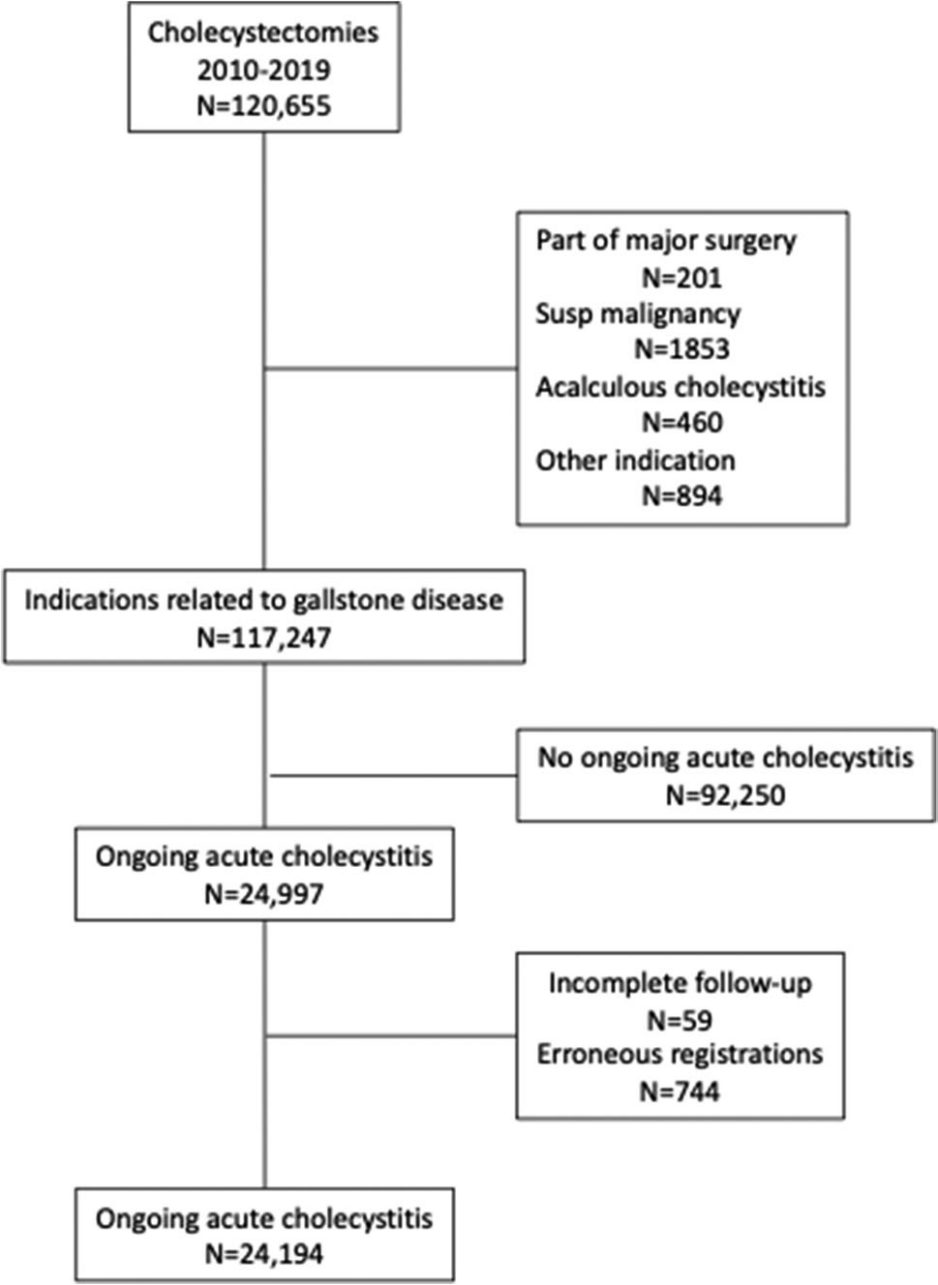
Flowsheet of included patients

This register-based cohort study analyzed data from the Swedish National Register for Gallstone Surgery and Endoscopic Retrograde Cholangiopancreatography (GallRiks). The cohort was defined as open or laparoscopic cholecystectomies on patients with AC between January 1, 2010, and December 31, 2019. Cholecystectomies performed for reasons other than gallstones (e.g., suspected malignancy or as part of a larger procedure) were excluded, as were planned cholecystectomies with the indication of biliary colic (Fig. 1). We also recorded whether the patient had a history of AC or not. Data on cholecystectomies included in the present study were derived from all hospitals performing acute surgery for AC.

### GallRiks

GallRiks is a nationwide quality register founded in May 2005 by the Swedish Society of Laparoscopic Surgery, the Swedish Society of Upper Abdominal Surgery, and the Swedish Surgical Association and covers over 90% of all cholecystectomies performed in Sweden.^10^ More than 13,000 cholecystectomies are included in the register each year, and about one-third are operated acutely.^11^ Registration is performed online by the surgeon or the theatre nurse during or immediately after surgery, and a 30-day mandatory follow-up is performed by a local coordinator at each participating unit. The local coordinator reviews the medical records of all patients and, if necessary, contacts the patient by phone to reduce the risk of missing postoperative adverse events. Patient characteristics, operation-specific parameters, and information about intra- and postoperative adverse events are recorded in GallRiks.

Adverse events were defined as any surgical, anesthesiologic, or cardiovascular complication associated with the cholecystectomy that could occur intra- or postoperatively, within 30 days.^12^

### Level of Care

In the context of this study, tertiary referral centers (TRCs) are hospitals that provide highly specialized surgery for malignancies such as pancreatic, liver, and esophageal cancer and perform benign surgeries like cholecystectomies. Due to the difficulty in distinguishing exactly between general and district hospitals, we merged these into one group — regional hospitals. The number of hospital beds at each surgical unit was obtained from the Swedish Association of Local Authorities and Regions. Unfortunately, the definition of a surgical unit varies somewhat between hospitals, with larger hospitals including not only general surgery but also sometimes other surgical specialties such as urology and orthopedics.

### Primary and Secondary Outcomes

The primary outcome was the number of days from admission to acute cholecystectomy in TRCs and regional units. The secondary outcome was to assess intra- and/or postoperative adverse event rates at the two hospital levels.

### Statistical Methods

Statistical analyses were carried out with JMP Pro (JMP®, Version 16.0.0 SAS Institute Inc., Cary, NC, USA). Pearson’s chi-square test was used to evaluate the differences between groups for dichotomous variables. Student’s *t*-test was used when comparing normally distributed numeric outcomes (e.g., age) between patients operated at TRCs and regional hospitals. Pearson’s linear correlation test was used to assess the association between the number of beds and the number of days between admission and surgery as well between days between admission and surgery and intra- and/or postoperative adverse events. Odds ratios of intra- and postoperative complications are given in the multivariable logistic regression analysis, adjusted for all possible confounders described in the baseline table (Table 1). A *p*-value of < 0.05 was regarded as statistically significant.

**Table 1:** Baseline characteristics and intra- and/or postoperative complications of the 24,194 included patients with acute cholecystitis

	Tertiary referral centers	Regional hospitals	*p*-value
Sex *n* (%) Female Male	3000 (51.1) 2873 (48.9)	9511 (51.9) 8810 (48.1)	0.2669
Age years (mean ± SD)	55.5 (16.3)	56.7 (16.4)	** < .0001**
ASA *n* (%) 1–2 > 2	4977 (84.7) 896 (15.3)	15,645 (85.4) 2676 (14.9)	0.2217
BMI *n* (%)* < 25 ≥ 25	608 (16.1) 3174 (83.9)	1871 (15.9) 9903 (84.1)	0.7866
Time to surgery days (mean ± SD) *n* (%) 1 day 2 days > 2 days	2.2 (1.7) 2276 (38.8) 1769 (30.1) 1828 (31.1)	1.6 (1.4) 10,347 (56.5) 4735 (25.8) 3239 (17.7)	** < .0001** ** < .0001** ** < .0001** ** < .0001**
Previous acute cholecystitis Yes No	591 (10.1) 5282 (89.9)	1624 (8.9) 16,697 (91.1)	**0.0056**

^*^8638 missing BMI

### Ethical Considerations

Ethics approval was granted by the Ethics Committee of Umeå University, Umeå, Sweden (DNR 2020–05,899, permit holder Lars Enochsson). Consent from the patients to participate in register-based research is required for registration in GallRiks. Patients are given the opportunity to withdraw all their personal data at any time from the register. Data extracted from the GallRiks register were anonymized, and statistical analyses were performed at the group level. The study was conducted in accordance with the Declaration of Helsinki.^13^

## Results

A total of 120,655 cholecystectomies were performed and included in the GallRiks register between January 1, 2010, and December 31, 2019. Of these, 24,194 (20%) cholecystectomies for AC met the inclusion and exclusion criteria (Fig. 1). Patient demographic data are presented in relation to hospital level in Table 1. Patients with a history of cholecystitis were more common at the TRC level compared with regional hospitals and were also slightly younger at TRC (Table 1). The distribution of sex, ASA, and BMI did not differ between TRCs and regional hospitals (Table 1). The difference between TRCs and regional hospitals in the frequency of patients with previous cholecystitis was limited to the second half of the study period (2015–2019) (10.2% for TRC vs. 8.2% for regional hospitals, *p* = 0.0003). Furthermore, time to surgery was significantly longer at TRCs compared with regional hospitals (2.2 ± 1.7 days vs. 1.6 ± 1.4 days, mean ± SD; *p* < 0.0001, Table 1).

**Fig. 2:**
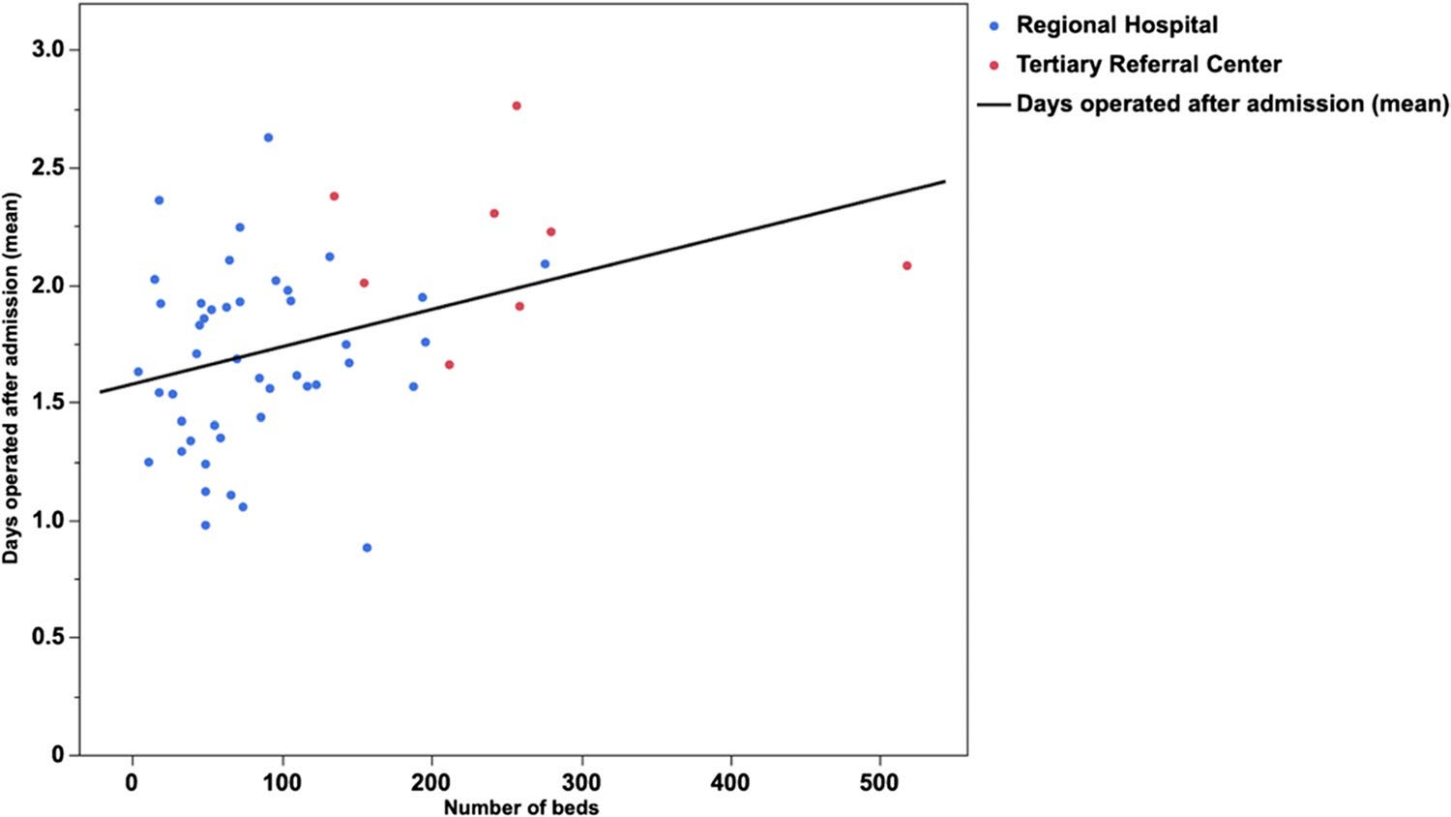
Linear fit of number of beds at surgical units in Sweden (*x*-axis) and days between admission and surgery (mean) on the *y*-axis (*R*^2^ = 0.132; *p* = 0.0075). TRCs (red dots) and regional hospitals (blue dots) are shown on the graph

**Fig. 3:**
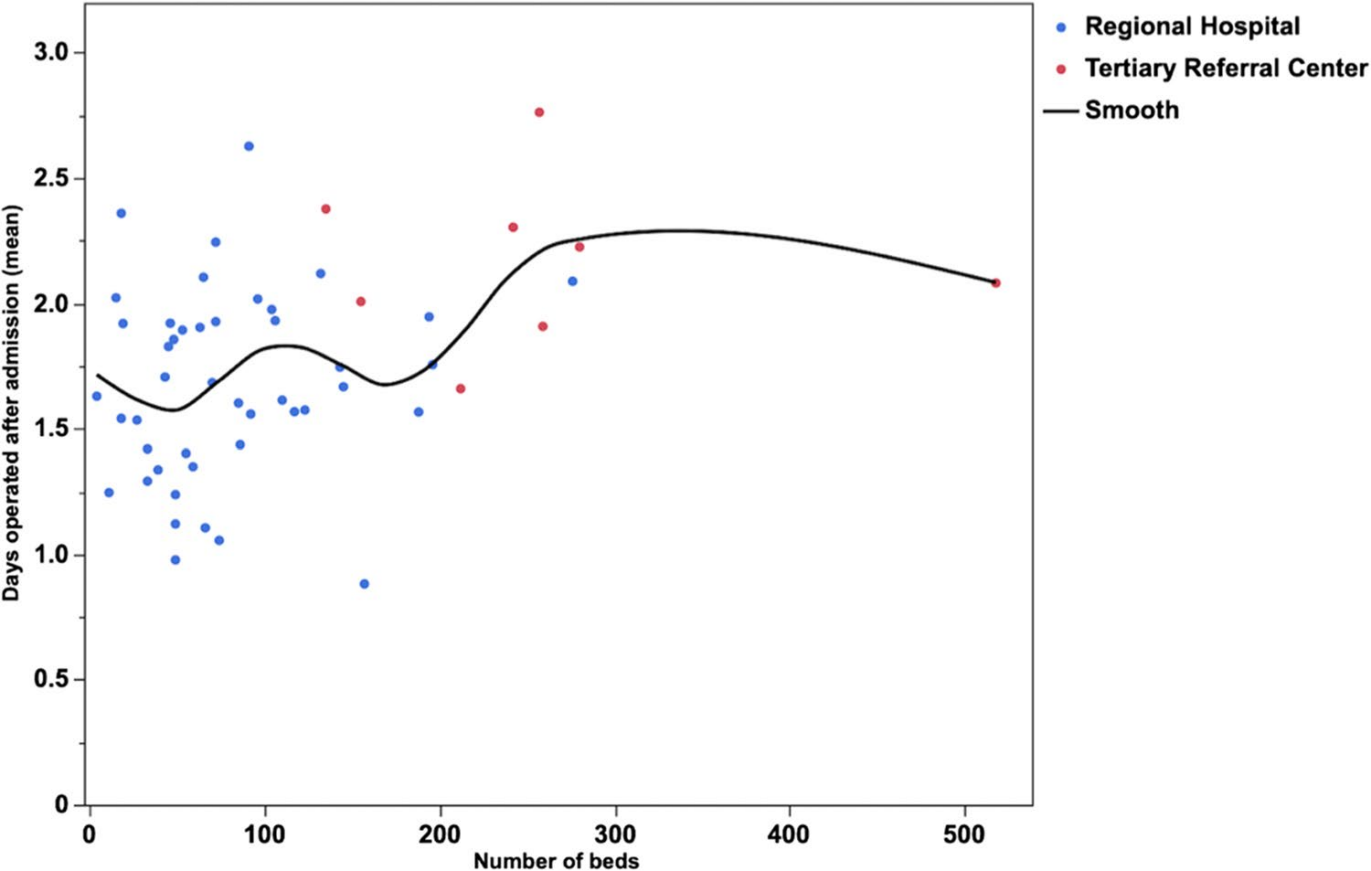
Spline graph showing number of beds at surgical units in Sweden on the *x*-axis and days between admission and surgery on patients with AC on the *y*-axis. TRCs (red dots) and regional hospitals (blue dots) are shown on the graph

**Fig. 4:**
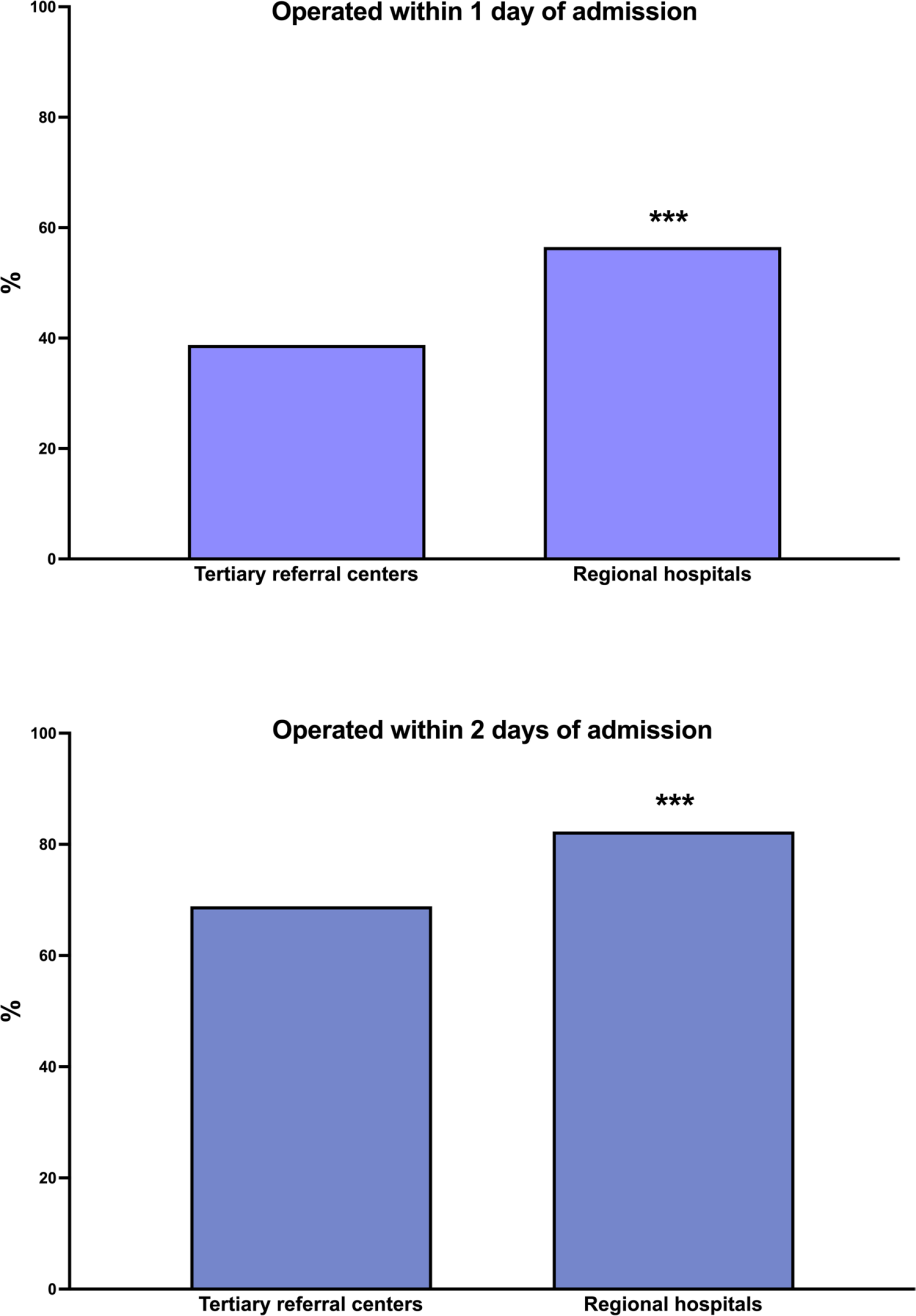
Bar chart showing the distribution between the proportion of patients with acute cholecystitis (%) operated on within 1 day (upper bar chart) and 2 days (lower bar chart), ***p < 0.0001

There was a moderate but significant correlation between the number of hospital beds and the time to surgery of patients with AC (*R*^2^ = 0.132; *p* = 0.0075, Fig. 2). However, the spline graph revealed that the process fluctuated significantly within different intervals on the *x*-axis (Fig. 3). When comparing TRCs with regional hospitals, data showed that 38.8% of patients admitted to a TRC with AC were operated on within 1 day of admission, whereas as much as 56.5% were operated on within the first day at regional hospitals (*p* < 0.0001, Table 1 and Fig. 4). When comparing proportions operated within 2 days the differences were somewhat less pronounced (Table 1). At TRCs, 31.1% of patients with ongoing AC did not undergo surgery until 2 days after admission to the hospital. Furthermore, at RH, more cholecystectomies were initiated and completed with the laparoscopic approach (78.6% vs. 74.3%; *p* < 0.0001).

**Fig. 5:**
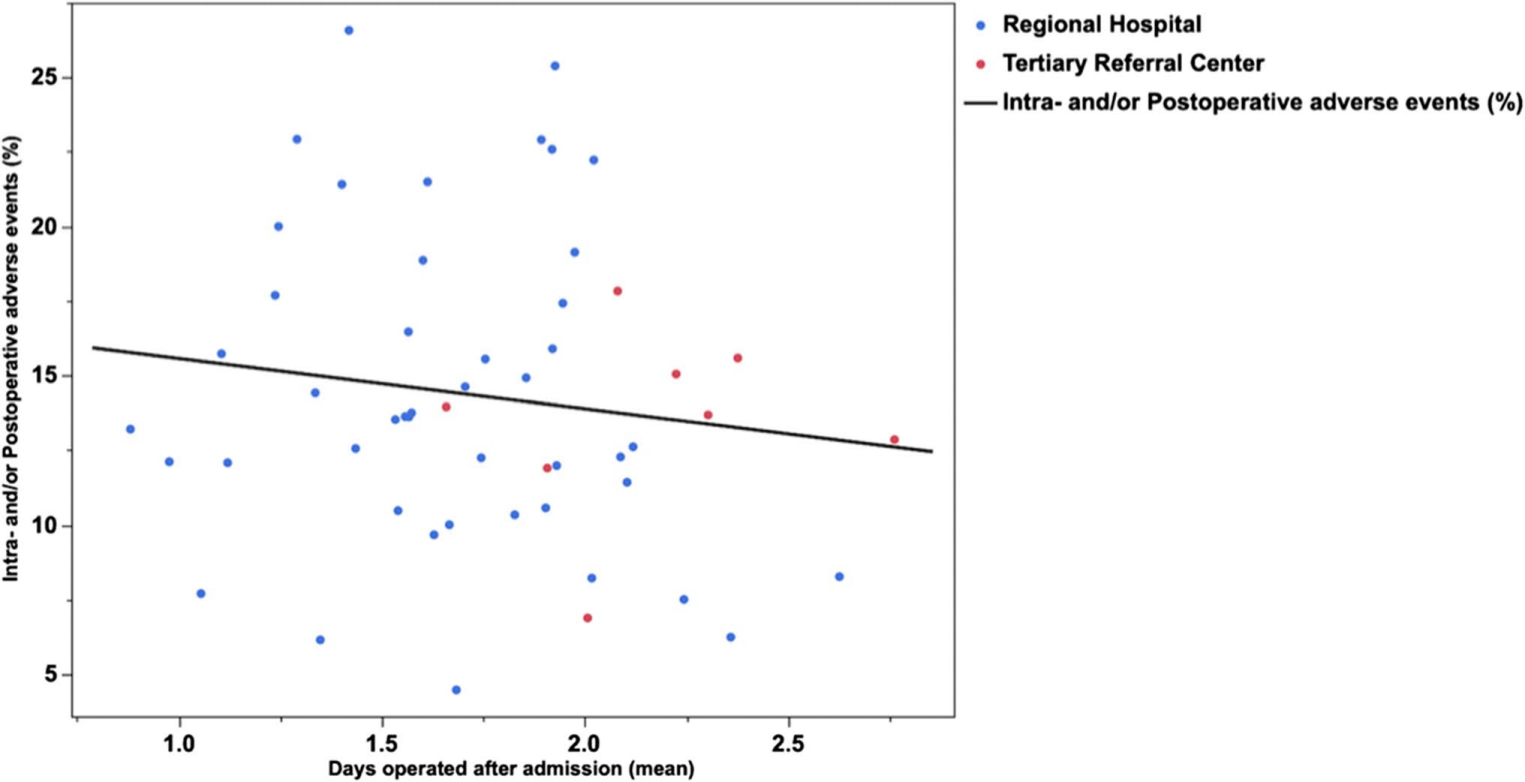
Linear fit of number of days between admission and surgery (mean) on the *x*-axis and intra- and/or postoperative complications (%) on the *y*-axis (*R*^2^ = 0.018; *p* = 0.3386). TRCs (red dots) and regional hospitals (blue dots) are shown on the graph

The number of hospital beds did not affect the rates of intra- and/or postoperative adverse events (*R*^2^ = 0.002; *p* = 0.7618), and there was no correlation between time to surgery and intra- and/or postoperative adverse events at the two hospital levels (Fig. 5). In regional hospitals, we noted that if the time to surgery in AC exceeded 2 days, there was a significant increase in the adverse event rate compared with if the patients were operated on within 2 days (15.3% vs. 13.5%; *p* = 0.0077). At TRCs, the percentages of the adverse events show a similar pattern, but not significant (14.4% vs. 13.4%; *p* = 0.3080), but perhaps since the number of patients operated at TRCs only constituted 24.3% of the patient material, there is not enough statistical power to answer this question. In the multivariable analysis (Table 2), the risk factors for intra- and/or postoperative adverse events were male sex, old age, ASA > 2, and previous AC. However, we did not find any differences in adverse event rates between the patients at TRCs and those at regional hospitals (Table 2).

**Table 2:** Odds ratio (OR) for intra- and/or postoperative adverse events within 30 days after acute cholecystectomy

	OR	95% CI	*p*-value
Sex Female Male	Ref 1.17	1.07–1.29	**0.0007**
Age < median ≥ median	Ref 1.59	1.44–1.75	** < .0001**
ASA 1–2 > 2	Ref 1.70	1.52–1.91	** < .0001**
BMI < 25 ≥ 25	Ref 0.94	0.83–1.06	0.3274
TRC Regional Hospital	Ref 1.01	0.91–1.13	0.8139
Time to surgery 1 day 2 days > 2 days	Ref 0.98 1.03	0.88–1.10 0.91–1.16	0.7823 0.6407
Previous AC No Yes	Ref 1.61	1.40–1.84	** < .0001**

Adjustments were made for variables included in the table

## Discussion

The present study shows that the time between admission and surgery for patients with AC is significantly longer at TRCs compared with regional hospitals in Sweden (Table 1; Fig. 4). An extended delay of more than 2 days negatively affected intra- and/or postoperative adverse event rates at the regional hospital level when analyzed using the chi-square test. We expected a similar trend at the TRC, but in the multivariable analysis, we did not find any significant differences between the two hospital levels regarding adverse events when adjusting for confounders (Table 2). Furthermore, this study shows that the number of hospital beds has a weak but significant correlation to the time until a patient with AC is operated on (Fig. 2). Surprisingly, it appears that the time to surgery after admission increases with the number of hospital beds in the surgical unit. However, the spline graph shows a fragmented pattern that is difficult to interpret (Fig. 3). Our interpretation is that when a unit becomes too large, there is a risk that prioritization of major cancer and emergency surgeries leads to a delay in benign surgeries such as cholecystectomy for AC. It is also worth noting that regional hospitals were significantly more likely to carry out the cholecystectomies with the laparoscopic technique. It is also possible that decisions are delayed if the surgical unit is too large and complex.

Significantly more patients operated at TRCs had previously had AC, which significantly increases the risk of adverse events. This indicates that more patients with AC are initially treated conservatively at TRCs and will be operated on electively at a later time.

Major procedures (e.g., esophageal, rectal, and pancreatic cancer surgery) performed at low-volume hospitals have higher adverse event rates than when performed at high-volume centers.^14,15^ Thus, many countries centralize these operations to high-volume TRCs.^16^ This is also true for liver resection, where patients undergoing the procedure at a high-volume center have higher postoperative survival rates, further supporting the centralization of major complex surgeries.^17,18^ While several studies have shown the positive effects of centralization on the outcome of major complicated surgeries, few studies have examined how centralization affects urgent yet benign surgery.

Since small hospitals cannot, and should not, perform complicated cancer surgery, these cases are referred to a TRC, thus freeing resources for acute benign surgeries to be performed at the regional hospital level. The risk is that this obligation to provide care for complicated patient groups at TRCs can delay less specialized benign surgeries or even referral to a regional hospital. This increases the time from admission to treatment and hence increases the risk for intra- and/or postoperative adverse events. Previous studies have shown a favorable impact on the outcome of surgery for AC when carried out soon after admission.^5,6^ This study also shows a beneficial outcome in regional hospitals if the patients are operated on within 2 days, which is in line with previous studies. However, there was no linear correlation overall between the number of days between admission and surgery and intra- and/or postoperative adverse events at the two hospital levels in the present study (Fig. 5). Furthermore, when adjusted for confounders, the multivariable analysis did not find any significant differences between TRCs and regional hospitals regarding complications.

The time between admission to surgery was significantly longer at the TRC level compared with regional hospitals (2.2 ± 1.7 days vs. 1.6 + 1.4 days; mean ± SD; *p* < 0.0001) (Table 1). This study focused on the treatment of patients with AC, where a cholecystectomy should be performed as soon as possible. It is concerning that there is a difference of up to 18% between TRCs and regional hospitals regarding how many patients with AC are operated on within 1 day of admission. It is also somewhat surprising that at TRCs, as many as 31.1% of the patients with ongoing AC waited for more than 2 days after hospital admission to receive an operation. The corresponding figure at regional hospitals is 17.7% (*p* < 0.0001) (Table 1). The consequence of the centralization of complicated malignant surgeries to TRCs may be that patients admitted with AC must wait longer for surgery or be conservatively treated with antibiotics and undergo surgery at a later stage. Indeed, 10.1% of patients operated for AC at a TRC had a history of cholecystitis compared with 8.9% of those operated at a regional hospital (Table 1). It is interesting that there was no difference between the two levels of hospital care regarding previous cholecystitis (9.9% vs. 9.8%; *p* = 0.8925) from 2010 to 2014, but from 2015 to 2019, the difference increased significantly (TRCs 10.2% vs. regional hospitals 8.3%; *p* = 0.0003). During the second half of the study, the centralization of highly specialized malignant surgery to TRCs was intensified which supports our hypothesis that centralization of malignant surgery can have negative side effects on benign surgery. Given that surgical adverse events result in longer hospital stays, whether delaying surgery for patients with AC has a negative effect on health economics is a worthwhile topic for future research.

A strength of this study is the large amount of prospectively collected data from the GallRiks register that covers over 90% of all cholecystectomies performed in Sweden, which minimizes the risk for selection bias.^10^ Nevertheless, there may have been a reporting bias. In a previous study, we found an association between the complication rate and the data completeness, which suggests that there may be selective underreporting.^19^ Another weakness is that patients with AC that undergo conservative treatment are not included in the register. At units where the capacity to provide timely surgery is impeded by the need to give priority to other patient groups, there may be a tendency to postpone surgery for acute cholecystitis. The data on history of previous AC managed conservatively is registered in GallRiks, which provides an indirect proof of the local and regional variations in the proportion of patients admitted with acute cholecystitis that are offered acute surgery.^7^

In conclusion, there is a slightly less risk of treatment delay at regional hospitals than at TRCs for patients with AC in Sweden. There was a discrete but significant correlation between the number of hospital beds in the surgical unit and when surgery was performed. The centralization of highly specialized surgeries to TRCs may have a beneficial to the outcome of malignant surgery, but the impact on benign surgery performed at these units has been unfavorable. In many cases, surgery for AC requires advanced technical skills, especially since one must operate soon after the onset of inflammation to achieve the best postoperative outcome, which the high frequency of adverse events in patients with a history of AC indicates. Benign surgery should ideally be concentrated to high-volume regional hospitals for the best results. The centralization of various types of surgery is common in most countries. It seems likely that these findings (i.e., increasing delay of benign surgery) are generalizable to other countries, but more studies are needed. International guidelines are required to secure equal standards of treatment regardless of where a patient is admitted. Although treatment inequality due to the uneven distribution of resources may be difficult to avoid entirely, guidelines could help to standardize the care of patients with gallstone disease.
